# Supplementary material for: Unraveling the plasticity of translation initiation in prokaryotes: Beyond the invariant Shine-Dalgarno sequence
Source: PLoS One. 2024 Jan 11;19(1):e0289914. doi: 10.1371/journal.pone.0289914 (PMC10783764; doi:10.1371/journal.pone.0289914)
Supplement: S1 Fig — The MEME matrix shows all the non-variable regions of the 16S rRNA, which commonly includes the CCUCC in the last motif. (PDF) [file pone.0289914.s005.pdf]

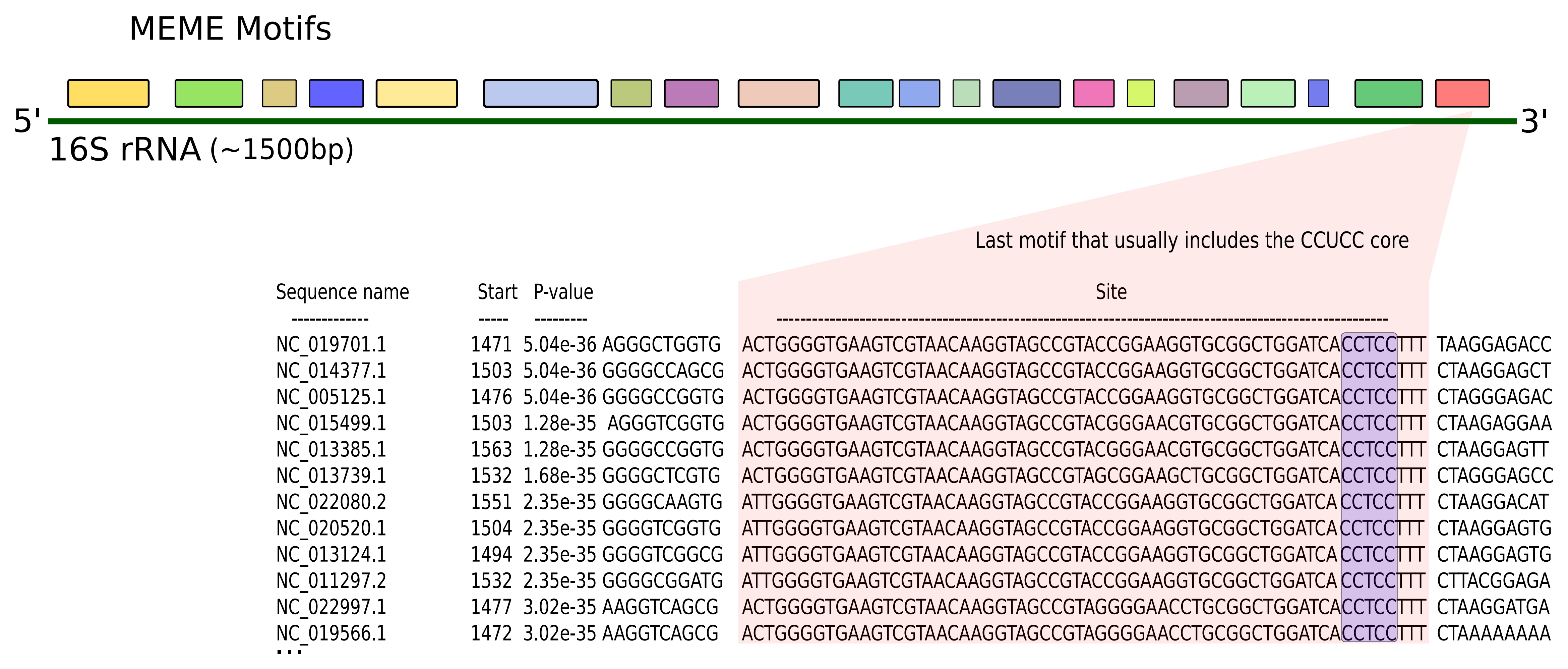
MEME Motifs
5'
3'
16S rRNA (~1500bp)
Last motif that usually includes the CCUCC core
Sequence name Start P-value Site
NC_019701.1 1471 5.04e-36 AGGGCTGGTG ACTGGGGTGAAGTCGTAACAAGGTAGCCGTACCGGAAGGTGCGGCTGGATCACCTCCTTT TAAGGAGACC
NC_014377.1 1503 5.04e-36 GGGGCCAGCG ACTGGGGTGAAGTCGTAACAAGGTAGCCGTACCGGAAGGTGCGGCTGGATCACCTCCTTT CTAAGGAGCT
NC_005125.1 1476 5.04e-36 GGGGCCGGTG ACTGGGGTGAAGTCGTAACAAGGTAGCCGTACCGGAAGGTGCGGCTGGATCACCTCCTTT CTAGGGAGAC
NC_015499.1 1503 1.28e-35 AGGGTCGGTG ACTGGGGTGAAGTCGTAACAAGGTAGCCGTACGGGAACGTGCGGCTGGATCACCTCCTTT CTAAGAGGAA
NC_013385.1 1563 1.28e-35 GGGGCCGGTG ACTGGGGTGAAGTCGTAACAAGGTAGCCGTACGGGAACGTGCGGCTGGATCACCTCCTTT CTAAGGAGTT
NC_013739.1 1532 1.68e-35 GGGGCTCGTG ACTGGGGTGAAGTCGTAACAAGGTAGCCGTAGCGGAAGCTGCGGCTGGATCACCTCCTTT CTAGGGAGCC
NC_022080.2 1551 2.35e-35 GGGGCAAGTG ATTGGGGTGAAGTCGTAACAAGGTAGCCGTACCGGAAGGTGCGGCTGGATCACCTCCTTT CTAAGGACAT
NC_020520.1 1504 2.35e-35 GGGGTCGGTG ATTGGGGTGAAGTCGTAACAAGGTAGCCGTACCGGAAGGTGCGGCTGGATCACCTCCTTT CTAAGGAGTG
NC_013124.1 1494 2.35e-35 GGGGTCGGCG ATTGGGGTGAAGTCGTAACAAGGTAGCCGTACCGGAAGGTGCGGCTGGATCACCTCCTTT CTAAGGAGTG
NC_011297.2 1532 2.35e-35 GGGGCGGATG ATTGGGGTGAAGTCGTAACAAGGTAGCCGTACCGGAAGGTGCGGCTGGATCACCTCCTTT CTTACGGAGA
NC_022997.1 1477 3.02e-35 AAGGTCAGCG ACTGGGGTGAAGTCGTAACAAGGTAGCCGTAGGGGAACCTGCGGCTGGATCACCTCCTTT CTAAGGATGA
NC_019566.1 1472 3.02e-35 AAGGTCAGCG ACTGGGGTGAAGTCGTAACAAGGTAGCCGTAGGGGAACCTGCGGCTGGATCACCTCCTTT CTAAAAAAAA
...
