# Supplementary figures and images for: Unraveling the plasticity of translation initiation in prokaryotes: Beyond the invariant Shine-Dalgarno sequence
Source: PLoS One. 2024 Jan 11;19(1):e0289914. doi: 10.1371/journal.pone.0289914 (PMC10783764; doi:10.1371/journal.pone.0289914)

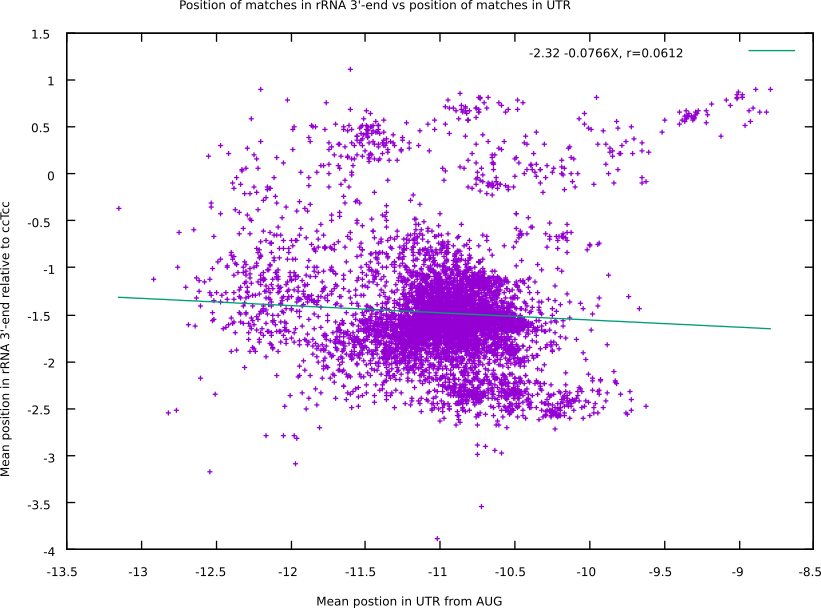

Supplement: S2 Fig — Linear regression indicates that the correlation is poor. (PNG) [file pone.0289914.s006.png]
